# Supplementary material for: PAIRUP-MS: Pathway analysis and imputation to relate unknowns in profiles from mass spectrometry-based metabolite data
Source: PLoS Comput Biol. 2019 Jan 14;15(1):e1006734. doi: 10.1371/journal.pcbi.1006734 (PMC6347288; doi:10.1371/journal.pcbi.1006734)
Supplement: S1 Fig — Data processing steps performed externally by Broad Metabolomics Platform or using existing software package (i.e. MICE) are highlighted in blue. Steps performed separately on data generated from each profiling method vs. on merged dataset containing data from all profiling methods are indicated by red text. All components of PAIRUP-MS were designed to be run in a streamlined manner, but each component can also be used independently depending on the characteristics of different datasets and analysis needs (see source code and documentation on https://github.com/yuhanhsu/PAIRUP-MS for details on input and output data format at each step). (PDF) [file pcbi.1006734.s001.pdf]

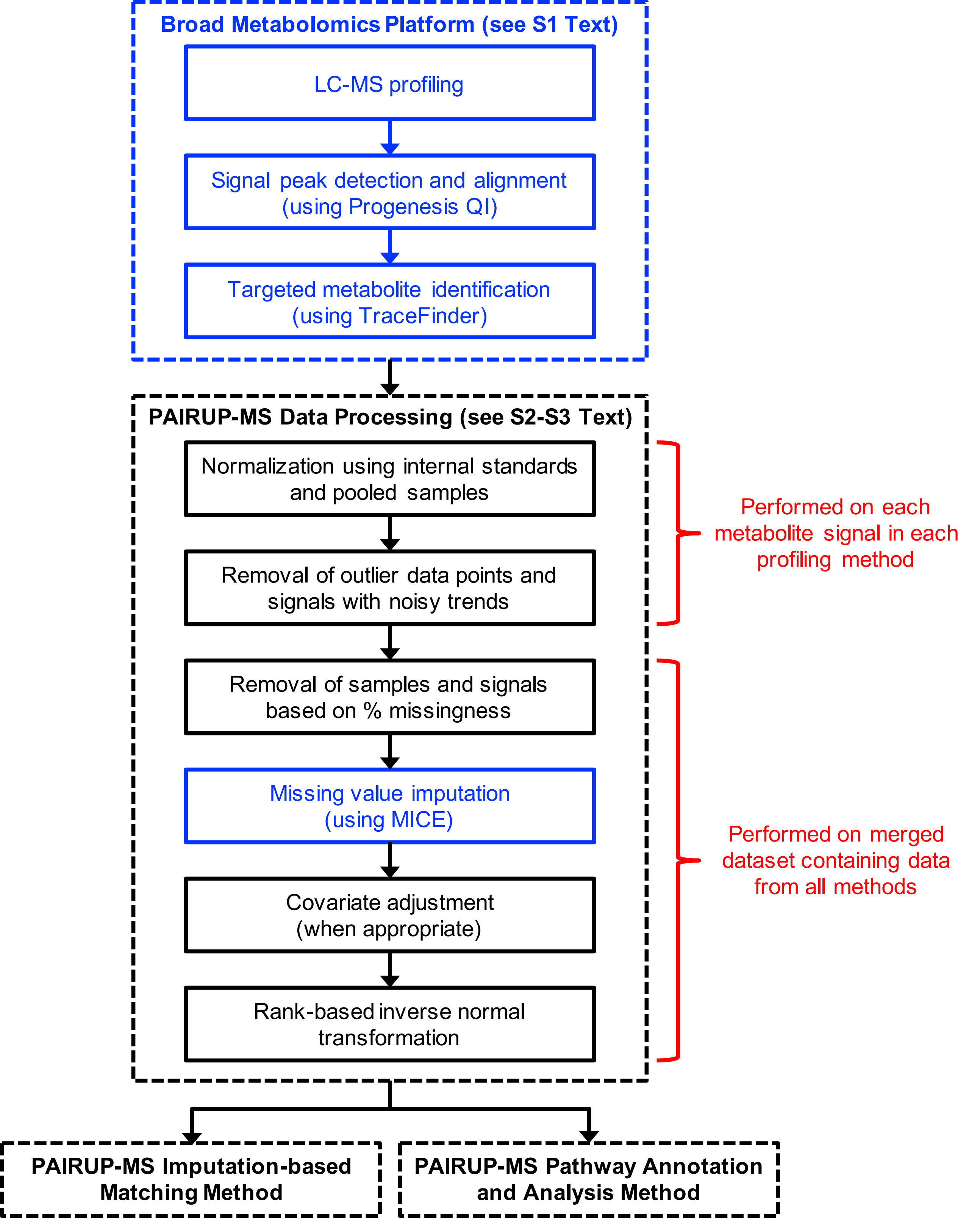
Broad Metabolomics Platform (see S1 Text)
LC-MS profiling
Signal peak detection and alignment (using Progenesis QI)
Targeted metabolite identification (using TraceFinder)
PAIRUP-MS Data Processing (see S2-S3 Text)
Normalization using internal standards and pooled samples
Removal of outlier data points and signals with noisy trends
Removal of samples and signals based on % missingness
Missing value imputation (using MICE)
Covariate adjustment (when appropriate)
Rank-based inverse normal transformation
Performed on each metabolite signal in each profiling method
Performed on merged dataset containing data from all methods
PAIRUP-MS Imputation-based Matching Method
PAIRUP-MS Pathway Annotation and Analysis Method
