# Supplementary material for: PAIRUP-MS: Pathway analysis and imputation to relate unknowns in profiles from mass spectrometry-based metabolite data
Source: PLoS Comput Biol. 2019 Jan 14;15(1):e1006734. doi: 10.1371/journal.pcbi.1006734 (PMC6347288; doi:10.1371/journal.pcbi.1006734)
Supplement: S2 Fig — Pairwise correlation between shared known metabolites were calculated within each dataset then compared across (a) OE vs. MCDS, (b) OE vs. BioAge, and (c) MCDS vs. BioAge. R-squared (Rsq) of the pairwise correlation values were calculated to assess overall similarity. (PDF) [file pcbi.1006734.s002.pdf]

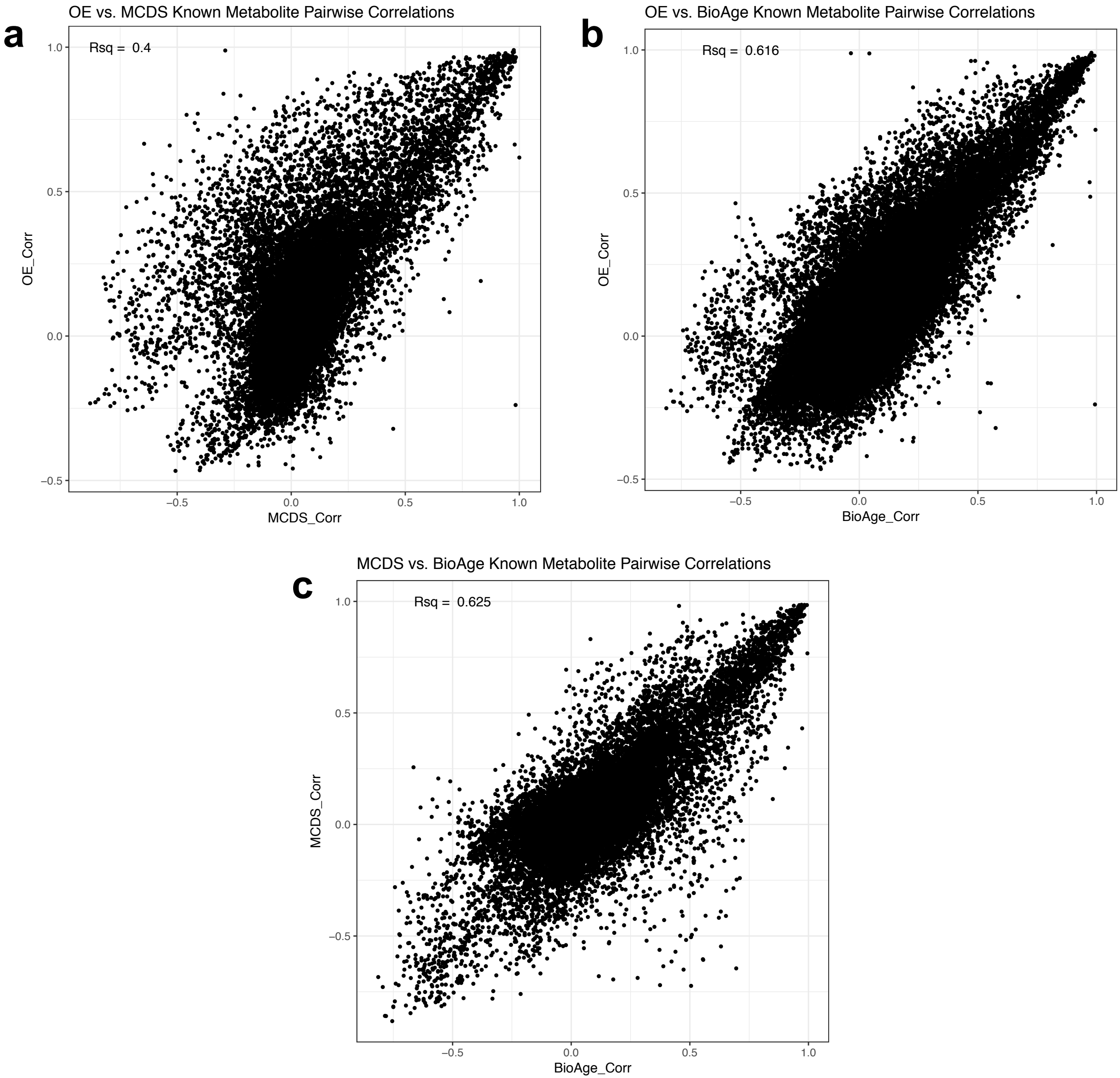
a
OE vs. MCDS Known Metabolite Pairwise Correlations
Rsq = 0.4
OE_Corr
MCDS_Corr
1.0
0.5
0.0
−0.5
−0.5
0.0
0.5
1.0
b
OE vs. BioAge Known Metabolite Pairwise Correlations
Rsq = 0.616
OE_Corr
BioAge_Corr
1.0
0.5
0.0
−0.5
−0.5
0.0
0.5
1.0
c
MCDS vs. BioAge Known Metabolite Pairwise Correlations
Rsq = 0.625
MCDS_Corr
BioAge_Corr
1.0
0.5
0.0
−0.5
−0.5
0.0
0.5
1.0
