# Supplementary material for: PAIRUP-MS: Pathway analysis and imputation to relate unknowns in profiles from mass spectrometry-based metabolite data
Source: PLoS Comput Biol. 2019 Jan 14;15(1):e1006734. doi: 10.1371/journal.pcbi.1006734 (PMC6347288; doi:10.1371/journal.pcbi.1006734)
Supplement: S5 Fig — Matching was performed using different adduct ion (a), correlation (b), match type (c), partition (d), and correlation cutoff (x-axis) settings (see Methods for parameter explanations). In each panel, the parameters not being compared were set to the following default values: “Combined”, “All Correlation”, “Reciprocal”, and “Within Method”. Dashed line in (b) shows optimal RT-based matching results for comparison. “Match Count”: number of shared knowns matched; “Correct Fraction”: fraction of correct matches; “Rsq0.8 Fraction”: fraction of matches strongly correlated (r2 > 0.8) with the correct known in observed data; “Incorrect Rsq0.8 Fraction”: fraction of incorrect matches strongly correlated with the correct known. (PDF) [file pcbi.1006734.s005.pdf]

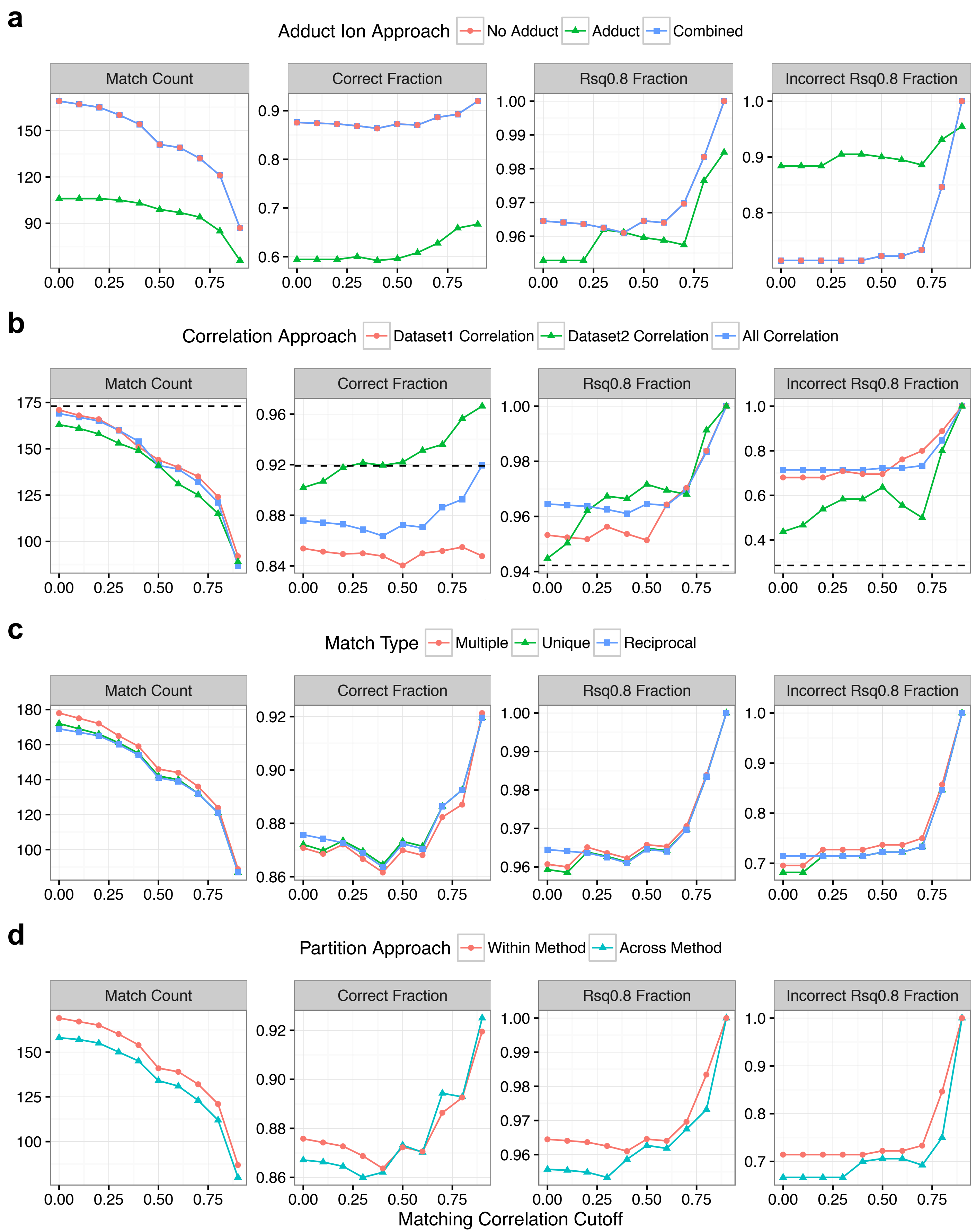

a
Adduct Ion Approach
No Adduct
Adduct
Combined
Match Count
Correct Fraction
Rsq0.8 Fraction
Incorrect Rsq0.8 Fraction
b
Correlation Approach
Dataset1 Correlation
Dataset2 Correlation
All Correlation
c
Match Type
Multiple
Unique
Reciprocal
d
Partition Approach
Within Method
Across Method
Matching Correlation Cutoff
