# Supplementary figures and images for: PAIRUP-MS: Pathway analysis and imputation to relate unknowns in profiles from mass spectrometry-based metabolite data
Source: PLoS Comput Biol. 2019 Jan 14;15(1):e1006734. doi: 10.1371/journal.pcbi.1006734 (PMC6347288; doi:10.1371/journal.pcbi.1006734)

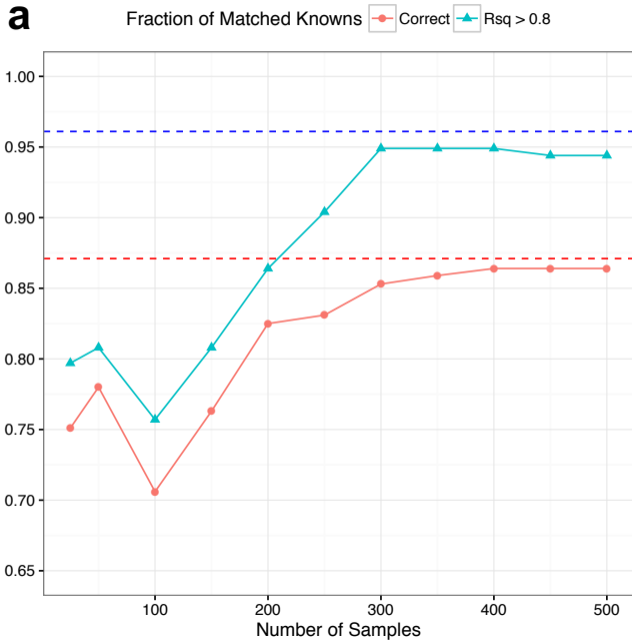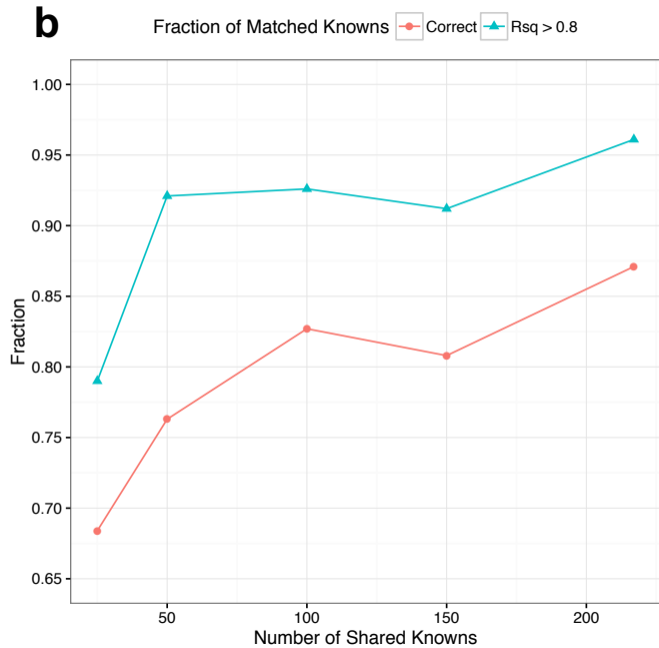

Supplement: S6 Fig — MCDS-BioAge matching was performed using (a) random subsets of MCDS and BioAge samples (and 217 shared known metabolites) or (b) random subsets of shared known metabolites (and 821 MCDS and 583 BioAge samples). For each matching analysis, the fraction of matched knowns that was correct (“Correct”) or highly correlated (“Rsq > 0.8”) with the correct match is plotted. In (a), “Number of Samples” indicates sample size per dataset; red and blue dashed lines indicate the correct and highly correlated fractions, respectively, when using all 821 MCDS and 583 BioAge samples to perform matching. Optimal MCDS-BioAge matching parameters shown in S2A Table were used for all analyses. (PDF) [file pcbi.1006734.s006.pdf]

Pair Type    Shared Known    Matched (reciprocal)    Matched (multiple)    Random

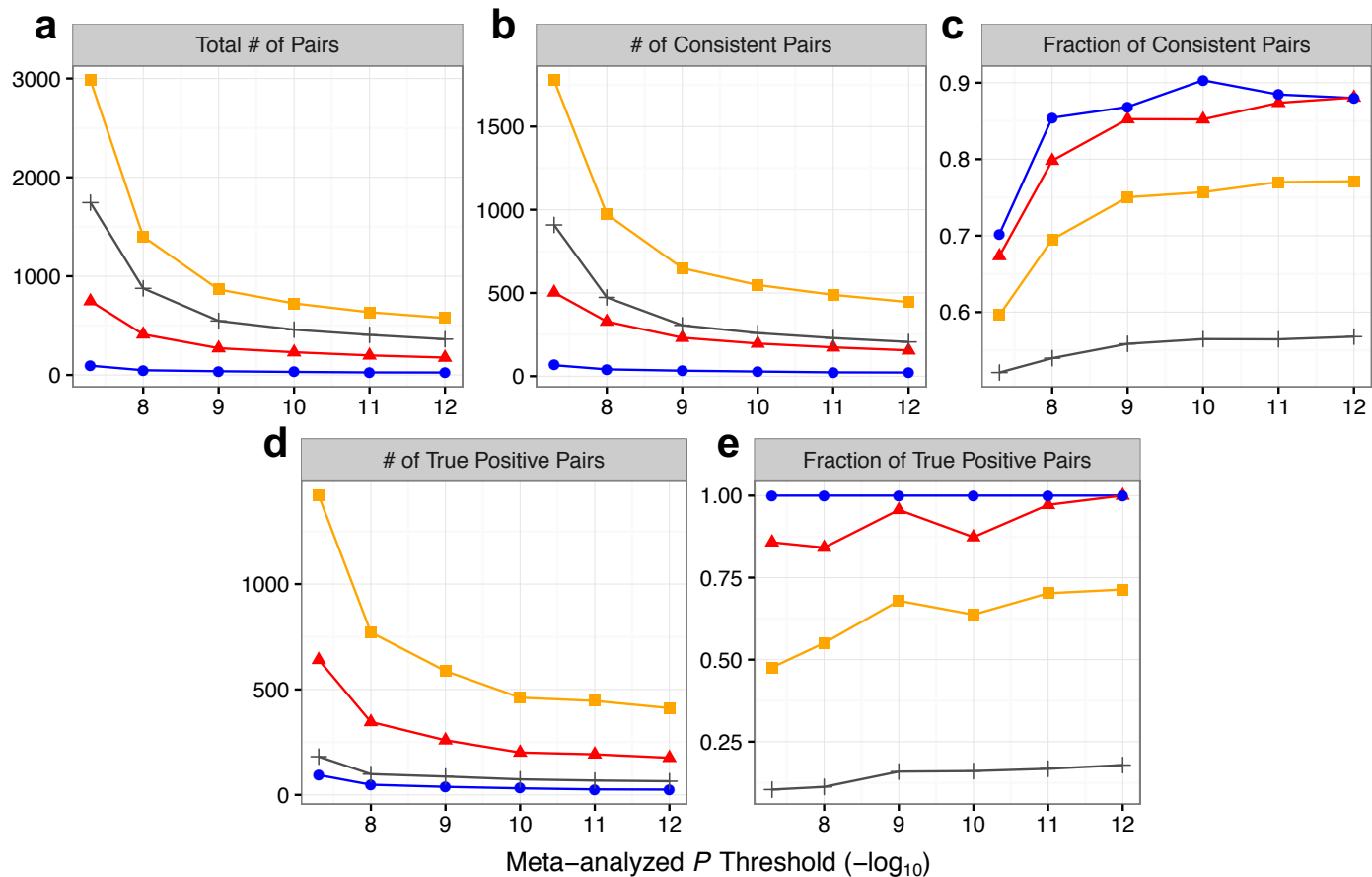

Supplement: S7 Fig — GWAS were performed for “multiple” matched signal pairs (a subset of which were “reciprocal”), shared known pairs (“Shared Known”, positive control), and randomly matched pairs (average statistics shown as “Random”, negative control) in OE and MCDS, followed by meta-analysis that ignored direction of effect. For each pair, the SNP with the best meta-analyzed p-value was selected to assess directional consistency of its association in the two cohorts. (a) “Total # of Pairs”: number of signal pairs with best SNPs below p-value threshold; (b) “# of Consistent Pairs” and (c) “Fraction of Consistent Pairs”: number and fraction of pairs with directionally consistent best SNPs below p-value threshold; (d) “# of True Positive Pairs” and (e) “Fraction of True Positive Pairs”: number and fraction of true positive pairs were estimated as described in Methods. X-axes start at genome-wide significant p-value threshold (p < 5 × 10^−8^). Error bars for “Random” pairs were excluded due to low visibility (all close to average). (PDF) [file pcbi.1006734.s007.pdf]

**a** Cumulative Variance and Cronbach's Alpha for MCs

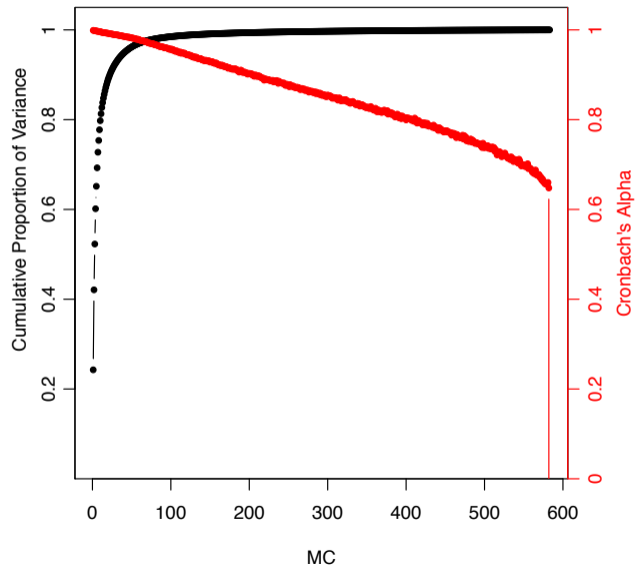

**b** Significant Metabolite Sets (5% FDR) per MC

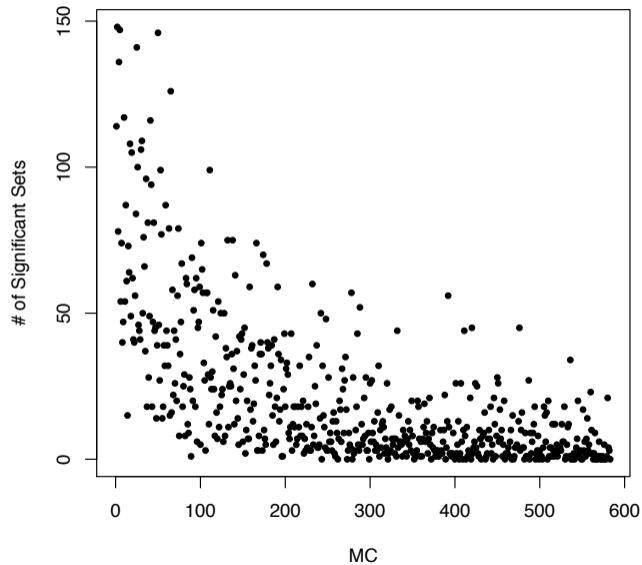

Supplement: S8 Fig — Principal component analysis was performed on the BioAge metabolite signal correlation matrix to derive the MCs. (a) Cumulative variance explained (left y-axis) or Cronbach’s alpha (right y-axis) of the MCs. (b) Number of CPDB metabolite sets that were enriched for each MC at 5% FDR. (PDF) [file pcbi.1006734.s008.pdf]

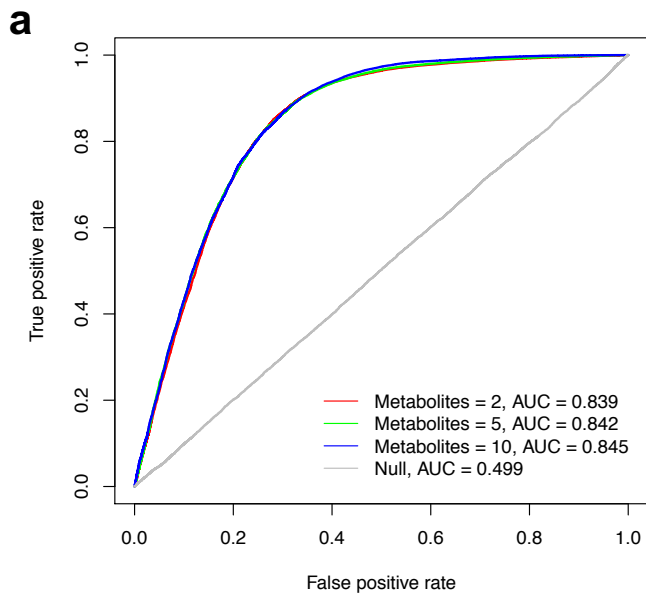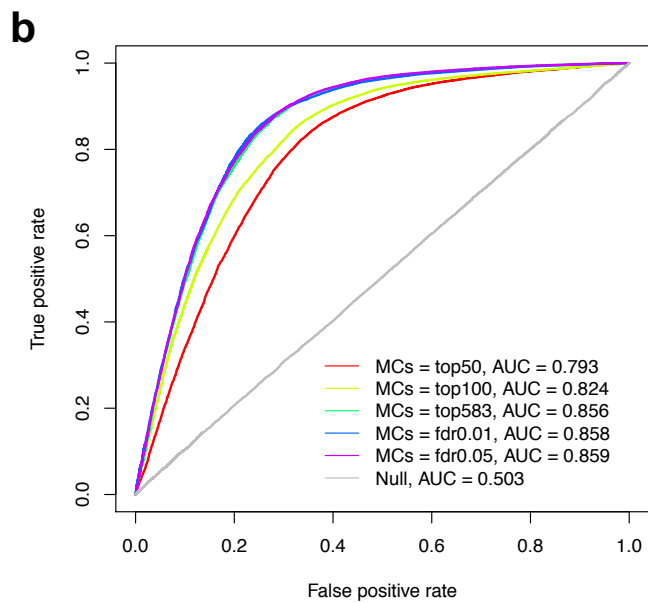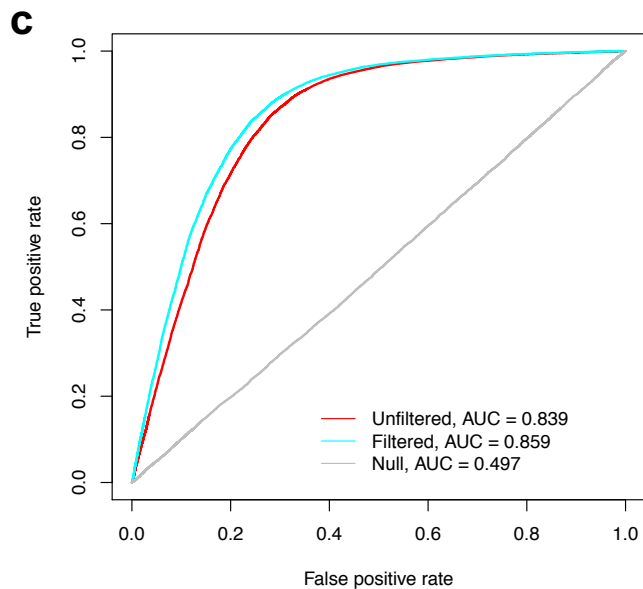

Supplement: S9 Fig — Annotation matrices generated using different combinations of (a) metabolite sets and (b) MCs were used to classify known metabolites into their original metabolite sets, generating a ROC curve (with corresponding area under the curve, AUC) for each matrix (see Methods for details). (c) We also compared an annotation matrix containing only the confidently reconstituted metabolite sets (i.e. label confidence score < 0.05; “Filtered”) against the full matrix (“Unfiltered”). In each panel, the parameters not being compared were set to the following default values: “Metabolites = 2” (i.e. metabolite sets containing > = 2 metabolites), “MCs = fdr0.05” (i.e. MCs enriched for > = 1 metabolite set at 5% FDR), and “Filtered”. “Null” statistics were calculated using permuted annotation matrix. (PDF) [file pcbi.1006734.s009.pdf]

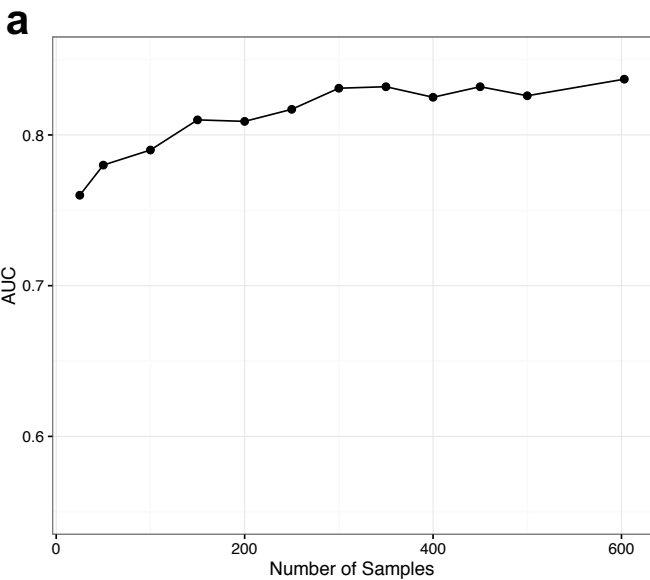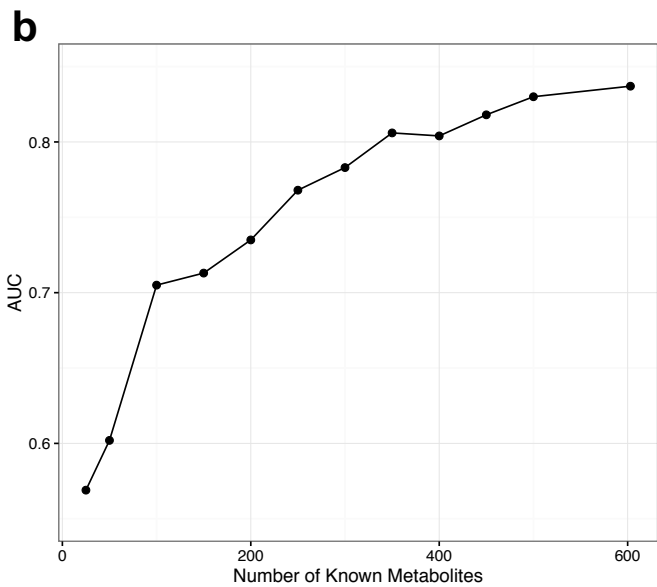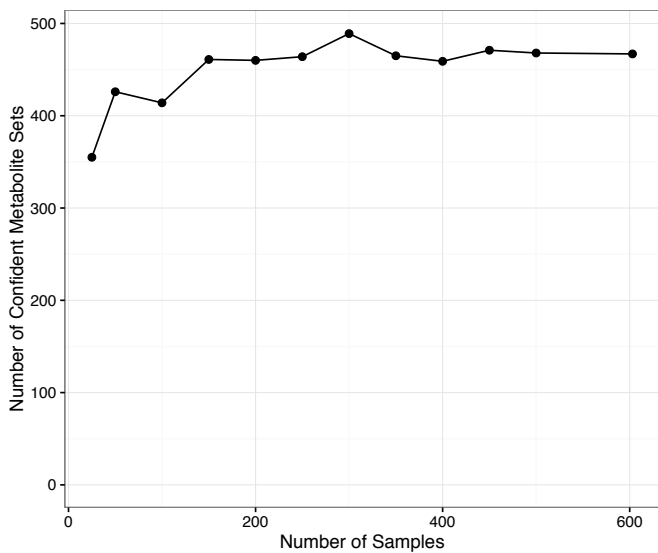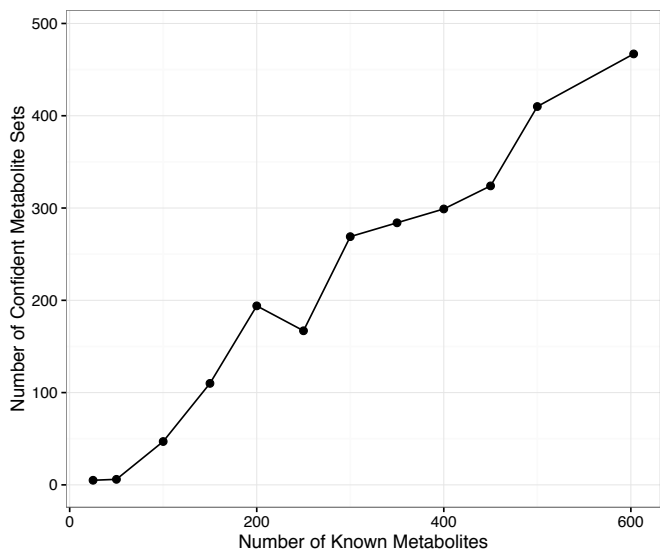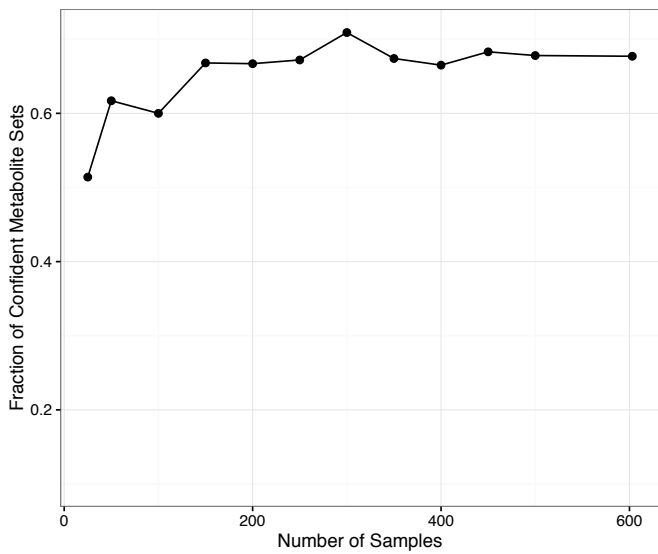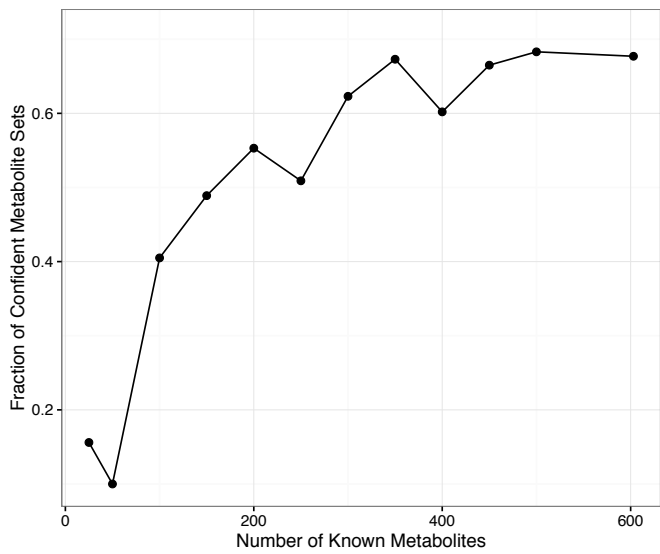

Supplement: S10 Fig — BioAge signal-metabolite set annotation matrices were generated using (a) random subsets of BioAge samples (and 603 known metabolites) or (b) random subsets of known metabolites (and 583 samples). For each resulting annotation matrix, the area under the curve (AUC) and the number or fraction of confidently reconstituted metabolite sets (i.e. label confidence score < 0.05) are plotted (see Methods for description of statistics). Metabolites sets containing > = 2 known metabolites and the top N MCs (N = sample size) were included for reconstitution in each analysis. (PDF) [file pcbi.1006734.s010.pdf]

**a****b****c**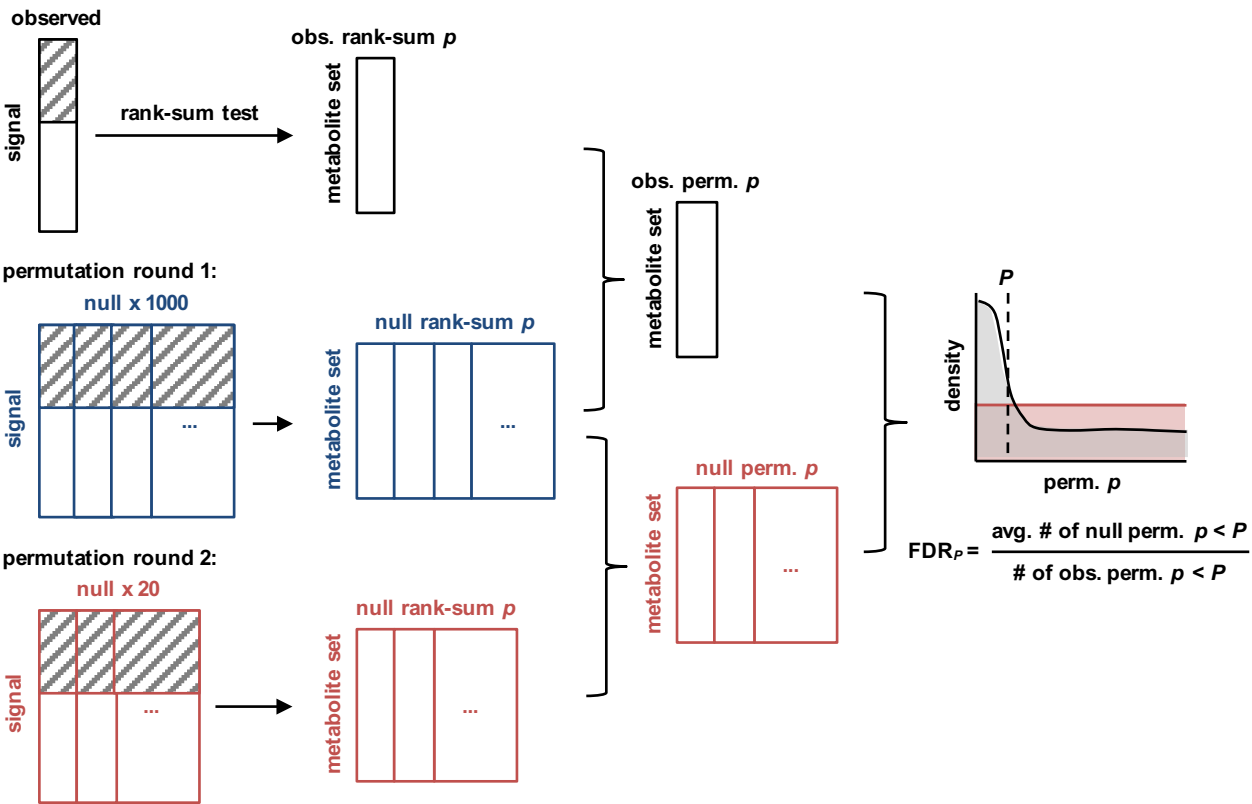

Supplement: S11 Fig — (a) Rank-sum p-value: Metabolite signals were split into positive (hatched pattern) and negative lists based on association with a variable of interest (e.g. BMI). The lists were used to perform two-tailed Wilcoxon rank-sum test to calculate observed (obs.) rank-sum p-value for each metabolite set. 2 rounds of null permutations (i.e. associating signals with randomly permuted variable) were used to calculate null rank-sum p-values. (b) Permutation p-value: Observed permutation (perm.) p-value for each metabolite set was calculated by comparing the observed rank-sum p-value against 1000 sets of null rank-sum p-values (from permutation round 1); 20 sets of null permutation p-values were calculated by comparing 20 sets of null rank-sum p-values (from permutation round 2) against another 1000 sets of rank-sum p-values (from permutation round 1). (c) FDR: The analysis-wide FDR for an observed permutation p-value threshold, P, was estimated by comparing the observed permutation p-values against the 20 sets of null permutation p-values. (PDF) [file pcbi.1006734.s011.pdf]
