# Supplementary material for: PAIRUP-MS: Pathway analysis and imputation to relate unknowns in profiles from mass spectrometry-based metabolite data
Source: PLoS Comput Biol. 2019 Jan 14;15(1):e1006734. doi: 10.1371/journal.pcbi.1006734 (PMC6347288; doi:10.1371/journal.pcbi.1006734)
Supplement: S2 Table — (a) Optimal parameter settings and corresponding calibration statistics for matching each pair of datasets. (b) Optimal parameter settings and corresponding calibration statistics for cross-method matching. (PDF) [file pcbi.1006734.s013.pdf]

S2 Table. (a) Optimal parameter settings and corresponding calibration statistics for matching each pair of datasets.

Matched datasets	Matching method	Parameter settings					# of matched signals	Shared known matching calibration statistics					
		Adduct ion	Correlation	Correlation cutoff	Partition	Match type		# of matches	# correct	# r^2 > 0.8	% correct	% r^2 > 0.8	% r^2 > 0.8 for incorrect
OE-MCDS	**IMP**	**Adduct**	**All correlation**	**None**	**Within method**	**Multiple**	**4432**[a,c]	**166**	**97**	**151**	**58.4**	**91.0**	**78.3**
						**Reciprocal**	**1573**[a,b,c]	**83**	**58**	**76**	**69.9**	**91.6**	**72.0**
	RT	Combined	N/A	N/A	Within method	Multiple	4432	166	94	151	56.6	91.0	79.2
						Reciprocal	1983	152	81	138	53.3	90.8	80.3
OE-BioAge	**IMP**	**Combined**	**Dataset 1 correlation**	**None**	**Within method**	**Multiple**	**7654**[d,e]	**252**	**216**	**237**	**85.7**	**94.0**	**58.3**
						**Reciprocal**	**3879**	**241**	**211**	**231**	**87.6**	**95.9**	**66.7**
	RT	Combined	N/A	N/A	Within method	Multiple	7654	252	215	224	85.3	88.9	24.3
						Reciprocal	3824	234	206	213	88.0	91.0	25.0
MCDS-BioAge	**IMP**	**Combined**	**All correlation**	**None**	**Within method**	**Multiple**	**4818**[e]	**178**	**155**	**171**	**87.1**	**96.1**	**69.6**
						**Reciprocal**	**2954**	**169**	**148**	**163**	**87.6**	**96.4**	**71.4**
	RT	Combined	N/A	N/A	Within method	Multiple	4818	178	163	168	91.6	94.4	33.3
						Reciprocal	3025	173	159	163	91.9	94.2	28.6

S2 Table. (b) Optimal parameter settings and corresponding calibration statistics for cross-method matching.

Matched datasets	Matching method	Parameter settings					# of matched signals	Shared known matching calibration statistics					
		Adduct ion	Correlation	Correlation cutoff	Partition	Match type		# of matches	# correct	# r^2 > 0.8	% correct	% r^2 > 0.8	% r^2 > 0.8 for incorrect
OE-MCDS	**IMP**	**Adduct**	**All correlation**	**None**	**Across method**	**Multiple**	**7911**	**170**	**94**	**155**	**58.9**	**88.4**	**71.8**
						**Reciprocal**	**1875**	**76**	**54**	**69**	**71.1**	**90.8**	**68.2**
	RT	Combined	N/A	N/A	Across method	Multiple	7911	170	75	142	44.1	83.5	70.5
						Reciprocal	2385	135	55	115	40.7	85.2	75.0
OE-BioAge	**IMP**	**Combined**	**Dataset 1 correlation**	**None**	**Across method**	**Multiple**	**9579**	**252**	**201**	**235**	**79.8**	**93.3**	**66.7**
						**Reciprocal**	**4041**	**240**	**195**	**228**	**81.3**	**95.0**	**73.3**
	RT	Combined	N/A	N/A	Across method	Multiple	9579	252	200	210	79.4	83.3	19.2
						Reciprocal	3994	223	187	194	83.9	87.0	19.4
MCDS-BioAge	**IMP**	**Combined**	**All correlation**	**None**	**Across method**	**Multiple**	**5251**	**178**	**148**	**170**	**83.1**	**95.5**	**73.3**
						**Reciprocal**	**3004**	**158**	**137**	**151**	**86.7**	**95.6**	**66.7**
	RT	Combined	N/A	N/A	Across method	Multiple	5251	178	141	146	79.2	82.0	13.5
						Reciprocal	3042	149	122	123	81.9	82.6	3.7

"# of matched signals": number of unknown or unshared known signals matched; "# of matches": number of shared known metabolites matched; "# correct" and "% correct": number and percentage of correct matches; "# r^2 > 0.8" and "% r^2 > 0.8": number and percentage of matches strongly correlated (r^2 > 0.8) with the correct known in observed data; "% r^2 > 0.8 for incorrect": percentage of incorrect matches strongly correlated with the correct known. "IMP": m/z and imputation-based matching; "RT": m/z and retention time-based matching; "N/A": not applicable. See Methods for detailed parameter explanations. Analyses that used the matching results: [a] matching validation; [b] GWAS replication and meta-analysis; [c] BMI-associated signal replication; [d] pathway validation; [e] BMI pathway analysis.
