# Supplementary material for: PAIRUP-MS: Pathway analysis and imputation to relate unknowns in profiles from mass spectrometry-based metabolite data
Source: PLoS Comput Biol. 2019 Jan 14;15(1):e1006734. doi: 10.1371/journal.pcbi.1006734 (PMC6347288; doi:10.1371/journal.pcbi.1006734)
Supplement: S4 Table — (PDF) [file pcbi.1006734.s015.pdf]

S4 Table. Calibration statistics for BioAge signal-metabolite set annotation matrix.

**Parameter settings**		**Calibration statistics**		
**Metabolites**	**MCs**	**AUC**	**# of confident sets**	**AUC for confident sets**
>= 2	top 50	0.772	469	0.793
>= 2	top 100	0.802	483	0.824
>= 2	top 583	0.837	467	0.856
>= 2	1% FDR (353)	0.840	488	0.858
**>= 2***	**5% FDR (541)***	**0.839**	**472**	**0.859**
>= 5	top 50	0.774	361	0.789
>= 5	top 100	0.806	366	0.821
>= 5	top 583	0.839	364	0.851
>= 5	1% FDR (351)	0.841	372	0.852
>= 5	5% FDR (531)	0.842	366	0.854
>= 10	top 50	0.769	245	0.781
>= 10	top 100	0.803	251	0.814
>= 10	top 583	0.839	252	0.847
>= 10	1% FDR (318)	0.838	251	0.846
>= 10	5% FDR (485)	0.845	250	0.853

"Metabolites": minimum number of metabolites required for including a metabolite set for reconstitution; "MCs": which MCs to use for reconstitution (see Methods), with number of MCs shown in parentheses for FDR-filtered MCs; "AUC": area under the ROC curve (generated by using annotation matrix values to classify known metabolites into their original metabolite sets); "confident sets": metabolite sets with label confidence scores (post-reconstitution rank-sum p) < 0.05. * Optimal parameter settings used for final BioAge annotation matrix, which yielded highest AUC for confident sets.
