# Supplementary material for: PAIRUP-MS: Pathway analysis and imputation to relate unknowns in profiles from mass spectrometry-based metabolite data
Source: PLoS Comput Biol. 2019 Jan 14;15(1):e1006734. doi: 10.1371/journal.pcbi.1006734 (PMC6347288; doi:10.1371/journal.pcbi.1006734)
Supplement: S5 Table — (PDF) [file pcbi.1006734.s016.pdf]

S5 Table. Summary of genetic validation of BioAge pathway annotations.

Significance threshold	# of loci with relevant enriched pathways			Empirical *p*
	Observed	Mean null	Median null	
0.05	13	6.8	6	0.1
0.025	10	3.35	2.5	0.05
0.01	5	0.85	1	< 0.05
0.005	4	0.3	0	< 0.05
0.001	2	0.5	0	< 0.05

"Significance threshold": permutation *p*-value threshold used to define metabolite set/pathway enrichment; "# of loci with relevant enriched pathways": number of loci in which the SNP-associated signals were enriched for >= 1 pathway linked to the affected gene. Mean and median null statistics and empirical *p*-values were calculated using 20 null permutations (see Methods).
