# Supplementary material for: PAIRUP-MS: Pathway analysis and imputation to relate unknowns in profiles from mass spectrometry-based metabolite data
Source: PLoS Comput Biol. 2019 Jan 14;15(1):e1006734. doi: 10.1371/journal.pcbi.1006734 (PMC6347288; doi:10.1371/journal.pcbi.1006734)
Supplement: S13 Table — (a) Rules for checking for m/z agreement between 2 ions. (b) m/z values of different adduct ions included in “adduct” settings. (PDF) [file pcbi.1006734.s024.pdf]

S13 Table. (a) Rules for checking for m/z agreement between 2 ions.

Ion Mode 1	Ion Mode 2	Setting	*d*
Positive	Positive	No adduct	0
Positive	Positive	Adduct	0 and pairwise m/z differences between the positive ions
Negative	Negative	No adduct	0
Negative	Negative	Adduct	0 and pairwise m/z differences between the negative ions
Positive	Negative	No adduct	m/z difference between $[M+H]^+$ and $[M-H]^-$
Positive	Negative	Adduct	m/z differences between each pair of positive and negative ions
Negative	Positive	No adduct	m/z difference between $[M-H]^-$ and $[M+H]^+$
Negative	Positive	Adduct	m/z differences between each pair of negative and positive ions

S13 Table. (b) m/z values of different adduct ions included in "adduct" settings.

Ion Mode	Ion	**m/z value** [1]
Positive	M^+	*m*
	$[M+H]^+$	*m* + 1.007276
	$[M+NH_4]^+$	*m* + 18.033823
	$[M+Na]^+$	*m* + 22.989218
Negative	$[M-H]^-$	*m* – 1.007276

m is the mass of the analyte molecule M. Additional or alternative adduct ions may be specified when running the m/z checking algorithm (see documentation on https://github.com/yuhanhsu/PAIRUP-MS).

References

1. Huang N, Siegel MM, Kruppa GH, Laukien FH. Automation of a Fourier transform ion cyclotron resonance mass spectrometer for acquisition, analysis, and e-mailing of high-resolution exact-mass electrospray ionization mass spectral data. Journal of the American Society for Mass Spectrometry. 1999;10(11):1166-73. doi: 10.1016/s1044-0305(99)00089-6.
