# Supplementary material for: PAIRUP-MS: Pathway analysis and imputation to relate unknowns in profiles from mass spectrometry-based metabolite data
Source: PLoS Comput Biol. 2019 Jan 14;15(1):e1006734. doi: 10.1371/journal.pcbi.1006734 (PMC6347288; doi:10.1371/journal.pcbi.1006734)
Supplement: S1 Text — (PDF) [file pcbi.1006734.s025.pdf]

S1 Text. Details of metabolite profiling.

All three metabolomics datasets used in this study were generated by the Metabolomics Platform at the Broad Institute.

OE and BioAge plasma samples were profiled using a combination of four nontargeted liquid chromatography tandem mass spectrometry (LC-MS) methods operated on systems comprised of Nexera X2 U-HPLC systems (Shimadzu Scientific Instruments; Marlborough, MA) coupled to Q Exactive or Exactive Plus orbitrap mass spectrometers (Thermo Fisher Scientific; Waltham, MA):

HILIC-pos**:** For positive ion mode analysis of polar metabolites, metabolite extracts were prepared by adding 90 µL of 74.9:24.9:0.2 v/v/v acetonitrile/methanol/formic acid containing stable isotope-labeled internal standards (valine-d8, Isotec; and phenylalanine-d8, Cambridge Isotope Laboratories; Andover, MA) to plasma (10 µL). The samples were centrifuged (10 min, 9,000 x g, 4°C) and the supernatants were injected directly onto a 150 x 2 mm Atlantis HILIC column (Waters; Milford, MA). The column was eluted isocratically at a flow rate of 250 µL/min with 5% mobile phase A (10 mM ammonium formate and 0.1% formic acid in water) for 1 minute followed by a linear gradient to 40% mobile phase B (acetonitrile with 0.1% formic acid) over 10 minutes. MS analyses were carried out using electrospray ionization in the positive ion mode using full scan analysis over m/z 70-800 at 70,000 resolution and 3 Hz data acquisition rate. Additional MS settings were: ion spray voltage, 3.5 kV; capillary temperature, 350°C; probe heater temperature, 300 °C; sheath gas, 40; auxiliary gas, 15; and S-lens RF level 40.

HILIC-neg**:** For negative ion mode MS analysis of polar metabolites, extracts were prepared by adding 120 µL of 80% methanol containing inosine-^{15}N4, thymine-d4 and glycocholate-d4 internal standards (Cambridge Isotope Laboratories; Andover, MA) to plasma (30 µL). The samples were centrifuged (10 min, 9,000 x g, 4°C) and the supernatants were injected directly onto a 150 x 2.0 mm Luna NH2 column (Phenomenex; Torrance, CA). The column was eluted at a flow rate of 400 µL/min with initial conditions of 10% mobile phase A (20 mM ammonium acetate and 20 mM ammonium hydroxide in water) and 90% mobile phase B (10 mM ammonium hydroxide in 75:25 v/v acetonitrile/methanol) followed by a 10 min linear gradient to 100% mobile phase A. MS analyses were carried out using electrospray ionization in the negative ion mode using full scan analysis over m/z 60-750 at 70,000 resolution and 3 Hz data acquisition rate. Additional MS settings were: ion spray voltage, -3.0 kV; capillary temperature, 350°C; probe heater temperature, 325 °C; sheath gas, 55; auxiliary gas, 10; and S-lens RF level 40.

C18-neg: For negative ion mode analysis of metabolites of intermediate polarity such as bile acids and free fatty acids, extracts were prepared by adding 90 µL of methanol containing PGE2-d4 as an internal standard (Cayman Chemical Co.; Ann Arbor, MI) to plasma (30 µL) and the samples were centrifuged (10 min, 9,000 x g, 4°C). The supernatants (10 µL) were injected onto a 150 x 2 mm ACQUITY T3 column (Waters; Milford, MA). The column was eluted isocratically at a flow rate of 400 µL/min with 25% mobile phase A (0.1% formic acid in water) for 1 minute followed by a linear gradient to 100% mobile phase B (acetonitrile with 0.1% formic acid) over 11 minutes. MS analyses were carried out using electrospray ionization in the negative ion mode using full scan analysis over m/z 200-550 at 70,000 resolution and 3 Hz data acquisition rate. Additional MS settings were: ion spray voltage, -3.5 kV; capillary temperature, 320°C; probe heater temperature, 300 °C; sheath gas, 45; auxiliary gas, 10; and S-lens RF level 60.

C8-pos: Plasma lipids were extracted by adding 190 µL of isopropanol containing 1,2-didodecanoyl-sn-glycero-3-phosphocholine as an internal standard (Avanti Polar Lipids; Alabaster, AL) to plasma (10 µL). Lipid extracts were centrifuged (10 min, 9,000 x g, ambient temperature) and supernatants (2 µL) were injected directly onto a 100 x 2.1 mm ACQUITY BEH C8 column (1.7 µm; Waters; Milford, MA). The column was eluted at a flow rate of 450 µL/min isocratically for 1 minute at 80% mobile phase A (95:5:0.1 vol/vol/vol 10 mM ammonium acetate/methanol/acetic acid), followed by a linear gradient to 80% mobile-phase B (99.9:0.1 vol/vol methanol/acetic acid) over 2 minutes, a linear gradient to 100% mobile phase B over 7 minutes, and then 3 minutes at 100% mobile-phase B. MS analyses were carried out using electrospray ionization in the positive ion mode using full scan analysis over m/z 200-1100 at 70,000 resolution and 3 Hz data acquisition rate. Additional MS settings were: ion spray voltage, 3.0 kV; capillary temperature, 300°C; probe heater temperature, 300 °C; sheath gas, 50; auxiliary gas, 15; and S-lens RF level 60.

All raw data were processed using Progenesis QI software (NonLinear Dynamics) for feature alignment, nontargeted signal detection, and signal integration. Targeted processing of a subset of known metabolites was conducted using TraceFinder 3.3 software (Thermo Fisher Scientific; Waltham, MA). Compound identities were confirmed using reference standards and reference samples.

MCDS plasma samples were profiled using three LC-MS methods that were similar to those used for the OE and BioAge cohorts, with some modifications as described below:

HILIC-pos and *LIPID*: Positive ionization mode analyses of polar metabolites and lipids were performed using an LC-MS system comprised of an Open Accela 1250 UHPLC coupled to a Q Exactive Orbitrap mass spectrometer (Thermo Fisher Scientific, Waltham MA). Samples for lipid analyses were prepared and separated using the C4 chromatography method described previously [1, 2] and MS data were acquired over m/z 400-1100.

CMH: Negative ionization mode analyses of polar metabolites were performed using a targeted LC-MS method as described previously [2, 3].

References

1. Rhee EP, Cheng S, Larson MG, Walford GA, Lewis GD, McCabe E, et al. Lipid profiling identifies a triacylglycerol signature of insulin resistance and improves diabetes prediction in humans. The Journal of clinical investigation. 2011;121(4):1402-11. doi: 10.1172/JCI44442. PubMed PMID: 21403394; PubMed Central PMCID: PMC3069773.
2. Townsend MK, Clish CB, Kraft P, Wu C, Souza AL, Deik AA, et al. Reproducibility of metabolomic profiles among men and women in 2 large cohort studies. Clinical chemistry. 2013;59(11):1657-67. doi: 10.1373/clinchem.2012.199133. PubMed PMID: 23897902; PubMed Central PMCID: PMC3812240.
3. Magnusson M, Lewis GD, Ericson U, Orho-Melander M, Hedblad B, Engstrom G, et al. A diabetes-predictive amino acid score and future cardiovascular disease. European heart journal. 2013;34(26):1982-9. doi: 10.1093/eurheartj/ehs424. PubMed PMID: 23242195; PubMed Central PMCID: PMC3703309.
