# Supplementary material for: PAIRUP-MS: Pathway analysis and imputation to relate unknowns in profiles from mass spectrometry-based metabolite data
Source: PLoS Comput Biol. 2019 Jan 14;15(1):e1006734. doi: 10.1371/journal.pcbi.1006734 (PMC6347288; doi:10.1371/journal.pcbi.1006734)
Supplement: S2 Text — (PDF) [file pcbi.1006734.s026.pdf]

S2 Text. Details of quality control (QC) of metabolite data.

To be able to assess the quality of a profiling run, the Broad platform adds an internal standard (IS) into each sample and two sets of control samples that are interspersed between the experimental samples in the analysis queue. One set of control samples is the pooled plasma (PP) samples that are randomly aggregated from the study samples; the other set of samples are commercial samples independent of the study to be analyzed. Both sets of control samples serve as technical replicates. Using these controls, we performed the following steps to normalize data generated by each profiling method: (1) removed signals with > 50% missingness in any quarter of the profiling run, (2) removed samples with outlier IS value, (3) normalized each sample to mean IS value, (4) removed signals for which the PP samples were all lower or higher than the study samples, (5) detected breakpoints (i.e. abrupt change in measurement over the course of a run) for each signal using forward and backward linear predictors and removed data points around a breakpoint, and (6) log-transformed and normalized each signal to cubic splines fitted to local PP samples (skipping over breakpoints).

After normalization, we performed several filtering steps to further reduce noise in the data: (1) removed signals for which the coefficient of variation (CV) of PP samples was > 10%, (2) removed data points > 4 standard deviations (SD) away from the mean of each signal, (3) removed windows (i.e. consecutive data points flanked by a pair of PP samples) with outlier mean or variance (> 3 SD away from mean window statistics), and (4) removed signals for which the variances across windows were found to be different by Levene's test.

To fine-tune the parameters used in the normalization and filtering procedures (e.g. number of flanking PP samples to use for fitting cubic spline or thresholds to use for identifying outlier data points and windows), we repeated the procedures using different settings and picked optimal parameters that generally (1) minimized variance of the commercial control samples (not available for BioAge), (2) minimized variation in window mean and variance, and (3) retained more known metabolites. Finally, we combined optimally QC'ed data from each method into one dataset and removed samples and signals with > 25% (OE) or > 50% (MCDS and BioAge) missingness.
