# Supplementary material for: PAIRUP-MS: Pathway analysis and imputation to relate unknowns in profiles from mass spectrometry-based metabolite data
Source: PLoS Comput Biol. 2019 Jan 14;15(1):e1006734. doi: 10.1371/journal.pcbi.1006734 (PMC6347288; doi:10.1371/journal.pcbi.1006734)
Supplement: S3 Text — (PDF) [file pcbi.1006734.s027.pdf]

S3 Text. Details of missing value imputation for metabolite data.

After QC, we imputed the remaining missing values in our data using the MICE (multivariate imputation by chained equations) R package (v2.25). In brief, MICE is an iterative algorithm that loops through each variable with missing data to predict its values using other correlated variables as predictors in a regression model. Typically, to account for uncertainty, the algorithm is run multiple times to impute a dataset and the resulting imputations are either all carried into downstream analysis or averaged into a single imputation. For our data, we took the median of the separate imputations to create the final imputed dataset.

In order to choose appropriate parameters for MICE (i.e. number of imputation runs, number of iterations per run, type of regression model, and number of predictors to use in the model), we simulated metabolite data in which a subset of metabolites had their lowest or random values set to missing, imputed these values using MICE with different parameter settings, and then calculated the correlation between the imputed and true values to evaluate performance of the parameters. In the end, we used 5 imputation runs and the Bayesian linear regression model for all of our datasets. For OE and BioAge, we used 10 iterations per run and the 50 best correlated signals as predictors for each signal; for MCDS, we used 15 iterations per run and the 70 best correlated signals as predictors. For a small subset of missing values that failed to be imputed by MICE (< 0.017% of all data), we replaced them with the median value of each signal.

Lastly, we compared the MICE imputed datasets against datasets generated by alternative approaches (i.e. no imputation, or imputing missing values to be the minimum or median of each signal) when identifying metabolite signals associated with BMI, and confirmed that MICE is a more flexible approach that can accommodate different missingness patterns, which can lead to improved power in downstream analysis.
