# Supplementary material for: PAIRUP-MS: Pathway analysis and imputation to relate unknowns in profiles from mass spectrometry-based metabolite data
Source: PLoS Comput Biol. 2019 Jan 14;15(1):e1006734. doi: 10.1371/journal.pcbi.1006734 (PMC6347288; doi:10.1371/journal.pcbi.1006734)
Supplement: S4 Text — (PDF) [file pcbi.1006734.s028.pdf]

S4 Text. Details of imputed genotype data.

OE: EGCUT genotyping and imputation using an Estonian reference panel have been described previously [1, 2]. Briefly, the OE samples analyzed in our study were genotyped using Infinium CoreExome-24 BeadChip Kit (n = 163) or Illumina HumanOmniExpress (n = 131), and their data were cleaned, phased, and imputed along with other EGCUT samples genotyped using the same platforms as described before [1]. We merged the two imputed datasets into one dataset and included genotyping platform as a covariate when performing GWAS.

MCDS: MCDS is part of the SIGMA Consortium, for which the participants have been genotyped using the Illumina OMNI2.5 array [3], exome sequencing [4], and/or exome array [5]. Genotype imputation of SIGMA samples have been described previously [5]. Briefly, we first divided the samples into two datasets based on genotyping platforms. Dataset 1 comprised of 4,478 samples that had been genotyped by exome chip and OMNI2.5 (MCDS: n = 66). Dataset 2 comprised of 3,732 samples genotyped by exome chip, OMNI2.5, and exome sequencing (MCDS: n = 571). For each dataset, we filtered for variants with MAF > 0.001, phased the data using SHAPEIT2 [6] (v2.5), and performed imputation using the 1000 Genomes reference panel (phase 3, release June 2014) and IMPUTE2 [7] (v2.3.2). Just as we did for OE, we merged the two imputed datasets into one dataset and included genotyping backbone (i.e. Dataset 1 or 2) as a covariate when performing GWAS.

References

1. Guo MH, Nandakumar SK, Ulirsch JC, Zekavat SM, Buenrostro JD, Natarajan P, et al. Comprehensive population-based genome sequencing provides insight into hematopoietic regulatory mechanisms. Proc Natl Acad Sci U S A. 2017;114(3):E327-E36. doi: 10.1073/pnas.1619052114. PubMed PMID: 28031487; PubMed Central PMCID: PMC5255587.
2. Mitt M, Kals M, Parn K, Gabriel SB, Lander ES, Palotie A, et al. Improved imputation accuracy of rare and low-frequency variants using population-specific high-coverage WGS-based imputation reference panel. Eur J Hum Genet. 2017;25(7):869-76. doi: 10.1038/ejhg.2017.51. PubMed PMID: 28401899; PubMed Central PMCID: PMC5520064.
3. SIGMA T2D Consortium, Williams AL, Jacobs SB, Moreno-Macias H, Huerta-Chagoya A, Churchhouse C, et al. Sequence variants in SLC16A11 are a common risk factor for type 2 diabetes in Mexico. Nature. 2014;506(7486):97-101. doi: 10.1038/nature12828. PubMed PMID: 24390345; PubMed Central PMCID: PMC4127086.
4. SIGMA T2D Consortium, Estrada K, Aukrust I, Bjorkhaug L, Burtt NP, Mercader JM, et al. Association of a low-frequency variant in HNF1A with type 2 diabetes in a Latino population. JAMA. 2014;311(22):2305-14. doi: 10.1001/jama.2014.6511. PubMed PMID: 24915262; PubMed Central PMCID: PMC4425850.
5. Mercader JM, Liao RG, Bell AD, Dymek Z, Estrada K, Tukiainen T, et al. A Loss-of-Function Splice Acceptor Variant in IGF2 Is Protective for Type 2 Diabetes. Diabetes. 2017;66(11):2903-14. doi: 10.2337/db17-0187. PubMed PMID: 28838971; PubMed Central PMCID: PMC5652606.
6. Delaneau O, Zagury JF, Marchini J. Improved whole-chromosome phasing for disease and population genetic studies. Nat Methods. 2013;10(1):5-6. doi: 10.1038/nmeth.2307. PubMed PMID: 23269371.
7. Howie B, Fuchsberger C, Stephens M, Marchini J, Abecasis GR. Fast and accurate genotype imputation in genome-wide association studies through pre-phasing. Nature genetics. 2012;44(8):955-9. doi: 10.1038/ng.2354. PubMed PMID: 22820512; PubMed Central PMCID: PMC3696580.
